# Quality assessment and anti-obesity activity of *Stellaria media* (Linn.) Vill

**DOI:** 10.1186/1472-6882-12-145

**Published:** 2012-09-03

**Authors:** Neerja Rani, Neeru Vasudeva, Surendra Kumar Sharma

**Affiliations:** 1 Department of Pharmaceutical Sciences, Guru Jambheshwar University of Science and Technology, Hisar, Haryana, 125001, India

**Keywords:** *Stellaria media*, Anti-obesity activity, α-amylase, Lyophilized juice, High-fat-diet

## Abstract

**Background:**

Obesity is recognized as a social problem, associated with serious health risks and increased mortality. Numerous trials have been conducted to find and develop new anti-obesity drugs through herbal sources to minimize side effects associated with the present anti-obesity drugs. The present study was designed to evaluate the quality control parameters, quantitative phytochemical analysis (total phenolic, total flavonoids and total saponin content), and the anti-obesity effect of lyophilized juice (LJ) of *Stellaria media* (Linn.) Vill. by employing *in vitro* and *in vivo* models.

**Methods:**

*In vitro* studies were performed to evaluate the inhibitory activity of LJ on pancreatic amylase and lipase. The *in vivo* pancreatic lipase activity was evaluated by measurement of plasma triacylglycerol levels after oral administration of lipid emulsion to swiss albino mice. Furthermore, the anti-obesity effect of LJ was assessed at two doses, 400 mg/kg and 900 mg/kg body weight in mice fed a high-fat-diet with or without LJ for 6 weeks.

**Results:**

The LJ inhibited pancreatic amylase and lipase activity *in vitro* and elevated plasma triacylglycerol level in mice. LJ suppressed the increase in body weight, retroperitoneal adipose tissue, liver weights and serum parameters *viz.,* total cholesterol, total triglyceride, LDL-cholesterol level at the dose of 900 mg/kg body weight of the mice fed with high fat diet. The total phenolic, flavonoid and saponin contents were found to be 0.26 mg/g, 1.4 mg/g and 1.19 μg/g respectively of LJ.

**Conclusion:**

The anti-obesity effects of LJ in high-fat-diet fed mice may be partly mediated through delaying the intestinal absorption of dietary fat and carbohydrate by inhibiting digestive enzymes.

## Background

Obesity is defined as excessive accumulation of body fat that may impair health. It has become a worldwide epidemic [1]. Obesity is known to be related to increase risks of coronary heart diseases, hypertension, non-insulin-dependent diabetes mellitus and certain type of cancer [2]. The major factor contributing to obesity is imbalance between energy intake and expenditure [3]. One most important strategy in the treatment of obesity includes the development of nutrient digestion and absorption inhibitors, in an attempt to reduce the energy intake through gastrointestinal mechanisms without altering any central mechanisms. Inhibition of digestive enzymes is one of the most widely studied mechanisms used to determine the potential efficacy of natural products as anti-obesity agents. The medicinal foods are known to have not only nutritive and taste values but also medicinal effects, and they are prescribed in various traditional preparations [4]. At present, the potential of natural products for the treatment of obesity is still largely unexplored and might be an excellent alternative strategy for the development of safe and effective anti-obesity drugs [5]. In course of search for a plant used as enzymes inhibitor, the investigation was directed towards the anti-obesity activity of LJ of *Stellaria media*.

*Stellaria media* (Linn.) Vill. (Caryophylaceae) commonly known as Chickweed, is a favourite salad herb widely distributed throughout the Himalayas upto an altitude of 4300 m [6]. The species is medicinal and edible, rich in vitamins, minerals, flavonoids, triterpenoids, Gamma-linolenic-acid, phenols and beta carotene. Various phytoconstituents *viz*; lipids [7], pentasaccharide [8], and triterpenoid [9] have been reported from this species. It has been used to treat various diseases because of many biological activities in traditional medicine, such as inflammations of the digestive, renal, respiratory and reproductive tracts. It also possesses diuretic, expectorant, antiasthmatic and antifeedant properties [10]. Its water is an old wives’ remedy for obesity [11]. However, the effect of this plant on the obesity has not yet been examined. Therefore, present work was undertaken to study the anti-obesity potential of LJ of *Stellaria media* employing various *in vitro* and *in vivo* assay systems.

## Methods

### Chemicals

Pancreatic α amylase, pancreatic lipase and glycyrrhizic acid were purchased from Sigma (Aldrich Co. St. Louis, MO, USA). Methanol (HPLC grade), casein, soluble starch, vitamin and mineral mixture, glyceryl trioleate, lecithin, sodium cholate and TES buffer were purchased from Hi-media. All other chemicals were of reagent grade.

### Animals

Male swiss albino mice (5 weeks old) were used for the *in vivo* models. The animals were housed for 1 week under a 12 h/12 h light/dark cycle in a temperature humidity-controlled room. The animals were given free access to food and water. After adaptation to the lighting conditions for 1 week, the healthy animals were used in the *in vivo* models. The experimental protocols were approved by the Institutional Animal Ethical Committee, Guru Jambheshwar University of Science and Technology, Hisar (Regn No 0436).

### Plant material

The fresh plant of *Stellaria media* (Linn.) Vill. was collected from the campus of Guru Jambheshwar University of Science and Technology, Hisar. The plant was taxonomically identified and authenticated by Dr. H.B. Singh, Head, Raw Materials Herbarium and Museum Division of National Institute of Science Communication and Information Resources. The voucher specimen has been deposited in the herbarium section of the Pharmacognosy Division, Department of Pharmaceutical Sciences, Guru Jambheshwar University of Science and Technology, Hisar for further reference.

### Preparation of lyophilized juice

500 g of fresh herb was taken into blender cup of a mixer and blended for 10 min to make fine slurry. The final slurry was filtered through muslin cloth. The filtrate was frozen and lyophilized (Alpha 2–4 LD Plus) at 5 μm Hg pressure at −50°C. The LJ was placed in a plastic bottle, and then stored at −20°C until used.

### Quality control parameters of LJ

The LJ was subjected to various quality control parameters according to Indian Pharmacopoeia [12] and WHO Guidelines [13]. The physico-chemical parameters *viz;* ash values, loss on drying, heamolytic activity; heavy metal analysis (Lead, Cadmium, Arsenic), microbial (*E. coli*, *Salmonella Sp., S. aureus)* and aflatoxin (B1 + B2 + G1 + G2) contamination were determined. The preliminary phytochemical screening was also performed for the presence of major phytochemical constituents in LJ according to standard methods [14].

### Quantitative analysis of LJ

#### Determination of total phenolic content

The total phenolic content of LJ was estimated using the Folin-Ciocalteu method adapted from Singleton and Rossi [15]. LJ (0.02 ml, 1 mg/ml) was oxidized with 0.25 ml of 10% (v/v) Folin-Ciocalteu’s reagent and neutralized by adding 1.25 ml of 20% sodium carbonate. The absorbance was measured at 685 nm after incubating at 40°C for 40 min. Results are expressed as mg/g of gallic acid.

#### Determination of total flavonoid content

The AlCI_3_ method adapted from Lamaison and Carnet [16] was used for the determination of the total flavonoid content of the LJ. 0.4 ml (10 mg/ml) of LJ was added to 2 ml of a solution of 2% AlCI_3._6H_2_O. After proper mixing, the mixture was incubated for 10 min at ambient temperature. The absorbance of the solution was read at 440 nm. Flavonoid contents are expressed in mg/g of quercetin.

#### Determination of total saponin content

For total saponin content determination 2 g of LJ was blended with 2 ml of concentrated NH_4_OH (37%) for 3 min. The pH of solution was adjusted to a pH 7.0 with H_3_PO_4_ and then 1 ml of 10% diastase was added. This was incubated at 37°C for 30 min, cooled to room temperature, and transferred to a 100 ml volumetric flask with CH_3_OH. The final extract was diluted to volume with additional CH_3_OH and filtered through Whatman No. 42 paper prior to analyses [17]. Before injection to HPLC column, extracts were filtered through a 0.45 μm membrane filter (Millipore, Bedford, USA).

Chromatographic analyses were carried out on the HPLC system (Agilent Technologies 1260 infinity) consisted of 1260 DVD-VL/ 1260 ALS/ 1260 Binary pump and UV/ visible detector. Separation of saponins was done using a Zorbax Eclise XDB-C18 (Analytical 4.6 250 mm ID, particle size 5 μm) column at 1.5 ml/min flow rate. Detection was made at 245 nm at 25°C. The analysis used 20 μl of a sample solution. The mobile phase consisted of methanol, water and acetic acid in the ratio of (60:34:6 v/v). The solvents were filtered and degassed prior to use. The glycyrrhizic acid was used as standard. Quantification of the saponin is expressed in μg/g of LJ and determined by a standard curve from a plot of the peak area and matching concentration of the standard solution.

### Measurement of α-amylase inhibitory activity

The activity was measured using the method reported by Xiao et al. [18] and Yoshikawa et al. [19] with slight modifications. Substrate solution was prepared by dissolving soluble starch (500 mg) in 25 mL of 0.4 M NaOH and heating for 5 min at 100°C. After cooling in ice H_2_O, the pH of solution was adjusted to 7 with 2 M HCl, and water was added to adjust the volume to 100 ml. LJ solutions were prepared by dissolving in acetate buffer (pH 6.5) to make 1, 2, 3, 4 and 5 mg/ml solutions. The substrate (40 μL) and LJ (20 μL) solutions were mixed in a micro plate well and the mixtures were pre-incubated at 37°C for 3 min. Then 20 μL of α-amylase solution (50 μg/ml) was added to each well, and the plate was incubated for 15 min. The reaction was terminated by adding 80 μL of 0.1 M HCL; then 200 μL of 1 mM iodine solution was added. The absorbance (Abs) was measured at 650 nm. Inhibitory activity was calculated as follows:

Inhibition (%) = {1 - (Abs2-Abs1) / (Abs4-Abs3) X100}

Where Abs 1 is the absorbance of incubated solution containing LJ, starch and amylase; Abs 2 is the absorbance of incubated solution containing LJ and starch; Abs 3 is the absorbance of incubated solution containing starch and amylase; Abs 4 is the absorbance of incubated solution containing starch.

### Measurement of pancreatic lipase inhibitory activity

Lipase inhibitory activity was measured according to the method of Han et al. [20] with slight modifications. Substrate solution was prepared by sonication (10 min in an ice bath) of a mixture of glyceryl trioleate (80 mg), lecithin (10 mg), and sodium cholate (5 mg) suspended in 9 ml of 0.1 M TES buffer (pH 7.0). LJ was dissolved in 0.1 M TES buffer to make 1, 2, 3, 4 and 5 mg/ml solutions. The substrate (20 μl) and sample solutions (20 μl) in microplate wells were preincubated for 3 min; then 10 μl of lipase solution (20 μg/ml) was added to each reaction mixture and incubated for 30 min at 37°C. The absorbance was measured at 550 nm using a microplate reader. Inhibitory activity (%) was calculated as follows:

Inhibition (%) = {1 - (Abs6-Abs5)/(Abs8-Abs7)X100}

Where Abs 5 is the absorbance of incubated solution containing LJ, substrate and lipase; Abs 6 is the absorbance of incubated solution containing LJ and substrate; Abs 7 is the absorbance of incubated solution containing substrate and lipase; Abs 8 is the absorbance of incubated solution containing substrate.

### Plasma triacylglycerol level after oral administration of lipid emulsion to mice

The animals were divided in three groups and deprived of food overnight. Test groups were orally administered lipid emulsion (5 ml/kg) with LJ (400 mg/kg) and (900 mg/kg) respectively. Positive control group was given lipid emulsion alone. The oil emulsion was prepared with 7 ml of olive oil, 93 mg of cholic acid and 7 ml of deionized water. Food was withdrawn during the test. Blood samples were collected from the ophthalmic venous plexus at 0, 1, 2, 3, 4 and 5 h using a heparinaized capillary tube, and centrifuged at 6300 rpm for 10 min. Plasma triacylglycerol levels were measured using a commercial triglyceride assay kit (Erba diagnostics).

### High-fat-diet induced obesity

Male swiss albino mice (5 weeks old) were acclimatized for 1 week, fed a high-fat diet (HFD) for 2 weeks and randomly divided into four groups matched for body weight [21]. Each group contained six animals (one animal per cage). Control group was fed normal diet; (g/100 g food : corn starch, 40; sugar, 10; vitamin mixture, 1; mineral mixture, 4; casein, 20; cellulose, 5; soybean oil, 17; methionine, 3; 433.2 Kcal/100 g). Test groups received LJ (400 mg/kg) and (900 mg/kg) along with high-fat diet for six weeks orally. Positive control group received only high-fat diet for six weeks. The composition of high-fat diet was; (g/100 g food): corn starch, 10; sugar, 10; lard, 40; vitamin mixture, 1; mineral mixture, 4; casein, 20; cellulose, 5; soybean oil, 7; methionine, 3. The Kcal of HFD was 583 Kcal/100 g. The total food intake by each group was recorded at least twice weekly, and the body weight of each mouse was recorded once weekly. At the end of the experiment, the blood was taken by venous puncture under anesthesia with diethyl ether, and the mice were then killed with an overdose of diethyl ether. Experiments were performed in a ventilated room. The serum was prepared and frozen at −80°C until analysis. The liver and retroperitonal adipose tissue were dissected and weighed. The triglyceride (TG), total cholesterol (TC), HDL-cholesterol, LDL-cholesterol was measured using Triglyceride E-Test and Total Cholesterol E-Test kits.

### Histopathological study

For histopathological studies livers of the scarified mice were dissected, removed, washed with normal saline and put in 10% formalin solution. The fixed specimens were then trimmed, washed and dehydrated in ascending grades of alcohol. The tissue specimens were cleared in xylene, embedded in paraffin, sectioned at 4–6 μ thickness, stained with Haematoxylin-Eosin ( H and E) [22].

### Statistical analysis

All the results were expressed as mean ± standard error of mean (SEM). The data of all the groups were analyzed using one-way ANOVA followed by Dunnett’s *t*-test using the software Instat 3.0. In all the tests, the criterion for statistical significance was p <0.05.

## Results

### Quality control parameters of LJ

The physicochemical parameters total ash, water soluble ash, and acid**-**insoluble ash were found to be 20.5, 12.5 and 8.65% w/w respectively. The percentage moisture content was found to be 9% w/w. The haemolytic activity was found to be 0.66 units/g. The preliminary phytochemical screening of the LJ indicated the presence of mainly carbohydrates, saponins, phenols, tannins and flavonoids. The Atomic Absorption Spectroscopy study showed the presence of cadmium, lead, arsenic in LJ but below the WHO permissible limits and therefore safe to use. LJ showed complete absence of *E. coli*, *Salmonella typhi*, and *Staphylococcus aureus*. Aflatoxin (B1 + B2 + G1+ G2) were found to be less than 5 ppb.

### Total phenolic, flavonoid and saponin content

The total phenolic content in LJ was found to be 0.26 mg/g in gallic acid equivalents. The content of flavonoids, in quercetin equivalents in mg/g of plant extract was found to be 1.4 mg/g. The concentration of total saponin in LJ was found to be 1.19 μg/g of LJ as quantified with HPLC.

### Effect of LJ on pancreatic α-amylase and lipase activity

**Figure 1:**
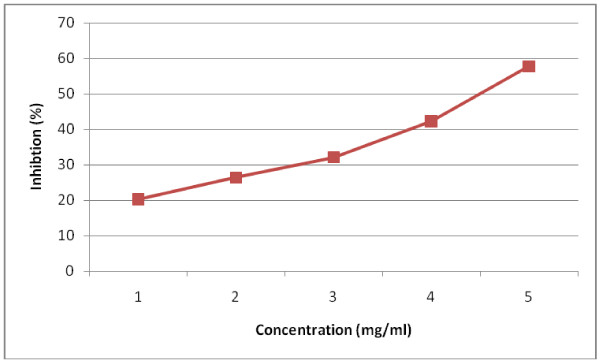
Inhibitory effect of LJ on α-amylase.

**Figure 2:**
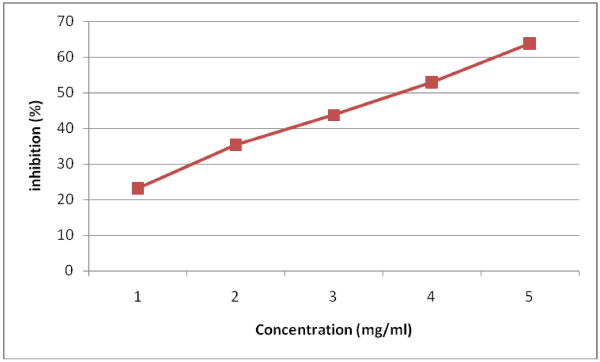
Inhibitory effect of LJ on Pancreatic Lipase.

The inhibitory activity of LJ against pancreatic α-amylase and lipase was determined using different concentrations (1, 2, 3, 4, 5 mg/ml)) of LJ. As shown in Figure 1. and Figure 2. LJ inhibited the enzyme activities in a dose-dependent way. The inhibition of lipase by LJ (IC_50_ value; 3.71 mg/ml) was stronger than that of α-amylase (IC_50_ value; 4.53 mg/ml).

### Effect of LJ on the plasma triacylglycerol levels after oral administration of lipid emulsion to mice

**Figure 3:**
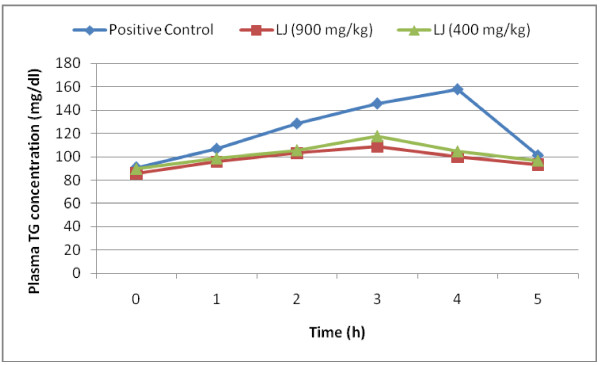
** Effect of LJ on elevation of the plasma triacylglycerol (TG) level after oral administration of a lipid emulsion.** Values are means ± SEM.

Figure 3. shows the serial changes in plasma triacylglycerol concentration when lipid emulsion with or without LJ was administered orally to mice. At 3 and 4 h after administration of LJ, the plasma triacylglycerol concentrations were significantly lower in group administered 900 mg/kg of LJ than those in the positive control group. There was no significant reduction in plasma triacylglycerol levels at the dose of 400 mg/kg body weight.

### Effect of LJ on food consumption, body, retroperitoneal adipose tissue, liver weight and serum parameters in mice fed a high-fat-diet for 6 weeks

The mean food consumption per week per mice was different between the control and high-fat-diet groups throughout the whole experimental period, but it did not differ between the groups fed high-fat-diet alone and high-fat-diet plus LJ treated group, suggesting that the anti-obesity effect of LJ was not mediated by a reduction of food intake (Table 1). The change in body weight of the groups during the experimental period of 6 weeks is shown in Table 1. The LJ at the dose of 900 mg/kg significantly suppressed the body weight gain when compared to the group fed on high-fat diet alone during experimental period. The oral administration of LJ to high-fat-diet induced obese mice for 6 weeks caused significant reductions in retropertonial adipose tissues and liver weight at the dose of 900 mg/kg body weight as compared to high-fat-diet. The LJ at the dose of 400 mg/kg body weight did not cause significant reduction in body weight, retropertonial adipose tissue, and liver weight [Table 1]. The serum concentrations of TG, cholesterol, and LDL-cholesterol were significantly lowered in the group fed high-fat-diet and treated with LJ at the dose of 900 mg/kg body weight, than in the control group fed on the high-fat-diet alone. The LJ at the dose of 400 mg/kg body weight did not cause significant reduction in serum parameters when compared to group receiving high-fat-diet alone [Table 2].

**Table 1:** ** Effect of LJ of* Stellaria media* on the body weight, food intake, parametrial fat and liver weight in mice fed on the high-fat-diet for 6 weeks orally**

**Group**	**Body weight gain (g)**	**Total food intake (kcal)**	**Retroperitoneal fat (g)**	**Liver weight (g)**
**(Kcal)**				
Control	2.81 ± 0.28	29.15 ± 0.10	0.98 ± 0.11	3.13 ± 0.11
HF diet	6.21 ± 0.75	41.97 ± 0.25	2.32 ± 0.37	4.45 ± 0.35
HF diet + LJ	5.36 ± 0.75	41.01 ± 0.73	1.59 ± 0.17	4.023 ± 0.24 (400 mg/kg)
HF diet + LJ	2.41 ± 1.35*	40.46 ± 0.63	0.92 ± 0.11**	3.58 ± 0.11* (900 mg/kg)

Data are expressed as mean ± SEM, n = 6, *P < 0.05, **P < 0.01 compared with high-fat-diet group.

**Table 2:** **Effect of LJ of*Stellaria media*on the blood parameters in mice**

**Serum parameter**	**Control**	**HF diet**	**HF diet + LJ**	**HF diet + LJ**
			**(400 mg/kg)**	**(900 mg/kg)**
Total triglyceride	88.72 ± 3.02	125.62 ± 5.70	110.50 ± 5.31	95.88 ± 3.96** (mg/dl)
Total cholesterol	132.23 ± 9.87	200.90 ± 17.34	181.47 ± 13.88	141.41 ± 12.03* (mg/dl)
HDL-cholesterol	68.06 ± 5.33	43.88 ± 3.41	51.8 ± 4.24	59.8 ± 3.99* (mg/dl)
LDL-cholesterol	56.16 ± 10.07	136.09 ± 15.97	111.23 ± 14.33	69.99 ± 9.13** (mg/dl)

Data are expressed as mean ± SEM, n = 6, *P < 0.05, **P < 0.01 compared with high-fat-diet group.

### Histopathological study

**Figure 4:**
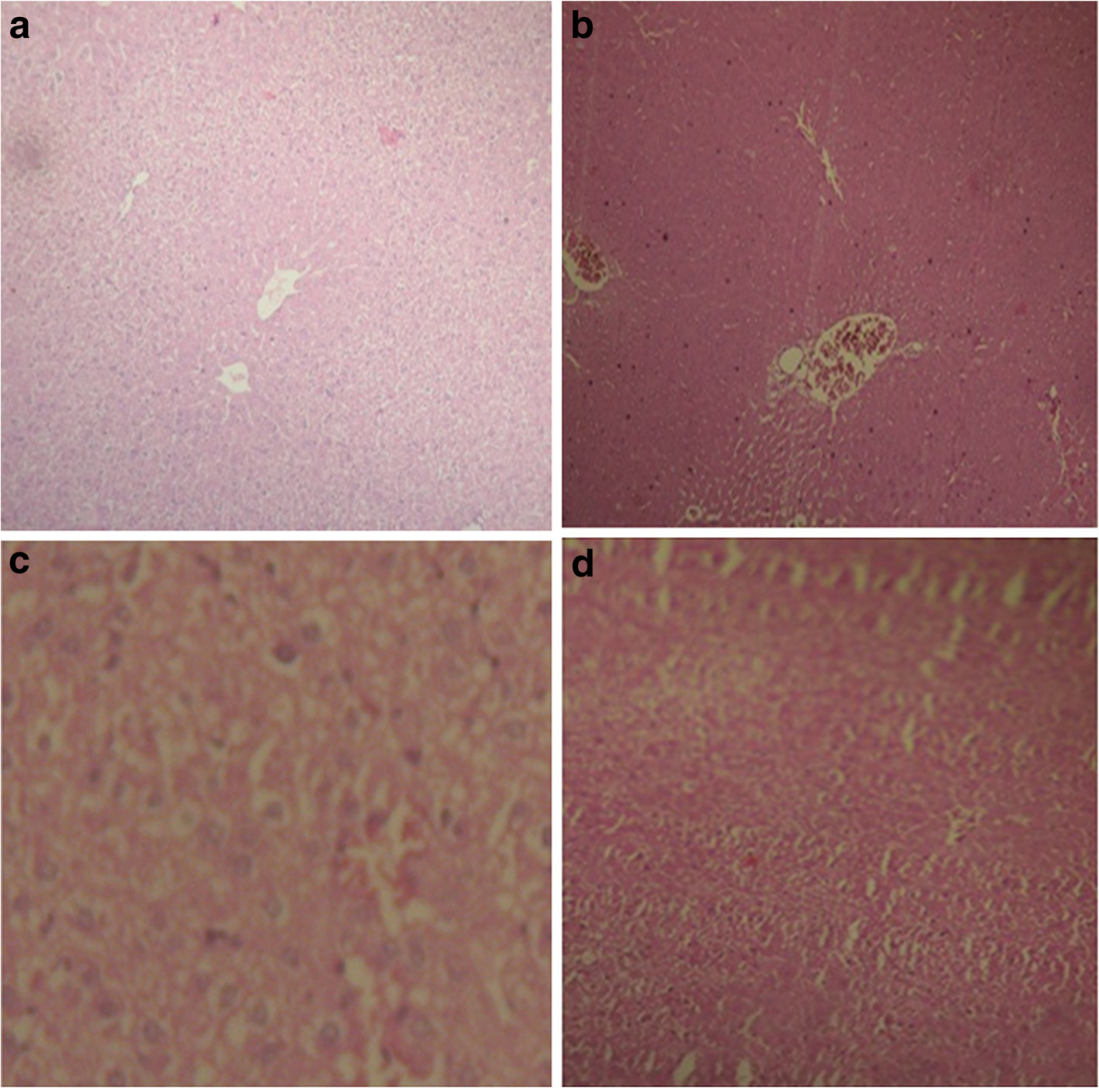
Effect of LJ on liver of mice (a) control (b) high-fat-diet (c) LJ treated group (900 mg/kg) (d) LJ treated group (400 mg/kg).

Histopathological examination of liver of the control group mice fed on normal diet revealed normal histological picture of hepatic lobule which consists of central vein surrounded by normal hepatocytes (Figure 4a). Examination of liver of mice fed on high-fat-diet showed fatty degeneration of hepatocytes and infiltration of leucocytes in hepatic sinusoid (Figure 4b). Liver of mice given orally LJ (900 mg/kg) showed marked improvement in fatty degeneration with no observed pathological lesions (Figure 4c). The LJ at the dose of 400 mg/kg body weight showed little vacuolar degeneration of hepatocytes and some improvement in fatty degeneration (Figure 4d).

## Discussion

Obesity is one of the major reasons for increase in incidence of coronary heart diseases, hypertension, non-insulin-dependent diabetes mellitus etc. A number of herbal extracts have been reported for their anti-obesity activities and are being used in Ayurveda for the same. *Stellaria media* is commonly used as salad herb for control of obesity but has not gained much importance as medicine due to lack of sustained scientific evidence. In the present study the effect of LJ of *Stellaria media* on digestive enzymes was assessed. *α-*amylase, one of the digestive enzyme secreted from the pancreas and salivary glands, is involved in an important biological process such as digestion of carbohydrates. Many crude drugs inhibit *α-*amylase activity [23]. Natural α*-*amylase inhibitors have been demonstrated to be beneficial in reducing post-prandial hyperglycemia by slowing down the digestion of carbohydrates and, consequently the absorption of glucose. Reducing post-prandial hyperglycemia prevent glucose uptake into adipose tissue to inhibit synthesis and accumulation of triacylglycerol [24]. On the other hand, it is well known that dietary lipid is not directly absorbed from the intestine unless it has been subjected to the action of pancreatic lipase. The two main products formed by the hydrolysis of pancreatic lipase are fatty acid and 2-monoacylglycerol. Based on these facts, inhibition of these digestive enzymes is beneficial in treatment of obesity. In *in vitro* studies LJ has exerted dose dependent inhibitory activity on *α-*amylase and pancreatic lipase. The inhibition of lipase by LJ (IC_50_ value; 3.71 mg/ml) was stronger than that of α-amylase (IC_50_ value; 4.53 mg/ml). the LJ has significantly decreased post-prandial triglyceride level at 3 and 4 h in *in vivo* studies at the dose of 900 mg/kg body weight. The study was continued to evaluate the effect of LJ on obesity in mice fed a high-fat-diet for six weeks. The administration of LJ at 900 mg/kg significantly suppressed the body weight of the mice. The inhibition of gain in body weight did not depend upon the decreased food or energy intake as there was no significant decrease in diet intake between the positive control and test groups, but was caused by preventing/delaying of fat and carbohydrate absorption. Long term feeding of LJ at the dose of 900 mg/kg to mice caused significant changes in blood parameters *viz;* decreased levels of total cholesterol, total triglyceride, and LDL- cholesterol, but an increased HDL-cholesterol level.

It has been demonstrated that tea saponin [25], saponin in *Platycodi Radix*[26,27] all belonging to the family of oleanene-type triterpenoid saponin; phenolic compounds [28] and flavonoids [29] showed strong inhibitory effects on pancreatic lipase and suppressed the increase of body weight induced by a high-fat-diet. *Stellaria media* is reported to possess oleanene-type triterpenoid saponin [9]. Our study quantified the presence of oleanene-type triterpenoid saponin, phenols and flavonoids in LJ which seems to be responsible for preventing high-fat-diet induced obesity.

## Conclusion

In conclusion, *Stellaria media* may prevent high-fat-diet induced fat storage in adipose tissue by inhibiting the intestinal absorption of dietary fat and carbohydrates through inhibition of digestive enzymes.

## Competing interests

The authors declare that they have no competing interests.

## Authors’ contributions

NR designed and planned the study; carried out experimental work, biochemical analysis, statistical analysis, interpretation and discussion of results related to their part of the work. SKS and NV designed and planned the study; drafted and revised the manuscript. NV checked and corrected the English language. All authors read and approved the final manuscript.

## Pre-publication history

The pre-publication history for this paper can be accessed here:

http://www.biomedcentral.com/1472-6882/12/145/prepub
